# Cancer-Associated Thrombosis: Pathophysiology, Laboratory Assessment, and Current Guidelines

**DOI:** 10.3390/cancers16112082

**Published:** 2024-05-30

**Authors:** Andreas G. Tsantes, Eleni Petrou, Konstantina A. Tsante, Rozeta Sokou, Frantzeska Frantzeskaki, Aglaia Domouchtsidou, Anastasios E. Chaldoupis, Sotirios P. Fortis, Daniele Piovani, Georgios K. Nikolopoulos, Nicoletta Iacovidou, Stefanos Bonovas, George Samonis, Argyrios E. Tsantes

**Affiliations:** 1 Laboratory of Haematology and Blood Bank Unit, “Attiko” Hospital, School of Medicine, National and Kapodistrian University of Athens, 12462 Athens, Greece; epetrou@med.uoa.gr (E.P.); dml23027@uniwa.gr (K.A.T.); achaldou@med.uoa.gr (A.E.C.); atsantes@med.uoa.gr (A.E.T.); 2 Microbiology Department, “Saint Savvas” Oncology Hospital, 11522 Athens, Greece; ldomouchtsidou@gmail.com; 3 Neonatal Intensive Care Unit, “Agios Panteleimon” General Hospital of Nikea, 18454 Piraeus, Greece; rosesok@med.uoa.gr; 4 2nd Department of Critical Care, Attikon Hospital, National and Kapodistrian University of Athens, 11527 Athens, Greece; ffrantzeska@gmail.com; 5 Laboratory of Reliability and Quality Control in Laboratory Hematology (HemQcR), Department of Biomedical Sciences, Section of Medical Laboratories, School of Health & Caring Sciences, University of West Attica (UniWA), 12243 Egaleo, Greece; sfortis@uniwa.gr; 6 Department of Biomedical Sciences, Humanitas University, Via Rita Levi Montalcini 4, Pieve Emanuele, 20090 Milan, Italy; daniele.piovani@humanitasresearch.it (D.P.); stefanos.bonovas@hunimed.eu (S.B.); 7 IRCCS Humanitas Research Hospital, Via Manzoni 56, Rozzano, 20089 Milan, Italy; 8 Medical School, University of Cyprus, 2029 Nicosia, Cyprus; nikolopoulos.georgios@ucy.ac.cy; 9 Neonatal Department, Aretaieio Hospital, National and Kapodistrian University of Athens, 11528 Athens, Greece; niakoid@med.uoa.gr; 10 Department of Medicine, University of Crete, 71500 Heraklion, Greece; samonis@med.uoc.gr; 11 Department of Oncology, Metropolitan Hospital, 18547 Athens, Greece

**Keywords:** cancer, malignancy, thrombosis, coagulopathy

## Abstract

**Simple Summary:**

Cancer-associated thrombosis (CAT) is a severe cause of mortality and morbidity in cancer patients, while active cancer is present in 20% of all patients with venous thromboembolism (VTE). CAT presents several peculiarities that distinguish this entity from other clinical settings associated with VTE. Management of thromboembolic events in CAT is extremely challenging because of the multifactorial pathophysiology of this clinical setting. The objective of this review is to enlighten the complicated pathophysiology of CAT, discuss the available biomarkers for early identification of high-risk patients, and summarize the current guidelines regarding the treatment and management of thrombotic events in cancer patients.

**Abstract:**

Dysregulated hemostasis in cancer patients is associated with various clinical conditions, from thromboembolic complications to disseminated intravascular coagulation. Despite the well-established association between cancer and thromboembolic complications, the mechanisms involved are not completely elucidated. There are several predisposing factors in cancer for increased thrombus generation, such as immobilization and chemotherapy. The term cancer-associated thrombosis (CAT) has been introduced to describe the close bidirectional relationship between cancer and thromboembolic events. Conventional coagulation tests (PT/aPTT) are more accurate in detecting a hypocoagulable rather than a hypercoagulable state; thus, their contribution to CAT management is limited. Traditionally, D-dimer levels have been the most common laboratory study for the evaluation of thrombotic risk. However, D-dimer levels only display a snapshot of the coagulation cascade, and they cannot provide a dynamic evaluation of evolving clot formation. Non-conventional assays, such as viscoelastic methods and microparticle formation are promising tools for the identification of patients at risk for developing CAT. Recent guidelines from the American Society of Clinical Oncology counsel against the estimation of thrombotic risk through a single test and recommend the use of scoring systems that take into account several risk factors. The present review outlines the current insights into the pathophysiological mechanisms of CAT and provides a comprehensive review of the latest advances in the laboratory assessment of CAT and the recent guidelines for the management of patients at risk for developing thromboembolic complications.

## 1. Introduction

Thrombosis is a common complication in patients with cancer and is associated with a high mortality rate. The association between malignancy and thrombotic events has been known since 1865 when Armand Trousseau first described the clinical association between thrombosis and undiagnosed cancer. Thus, the term Trousseau’s syndrome is often used to describe a thromboembolic event as the first symptom of a malignant disease [1]. Thrombotic complications in cancer patients vary from venous or arterial thromboembolism, to disseminated intravascular coagulation (DIC). The latter is usually observed in patients with hematological malignancies and metastatic disease, whereas venous thromboembolism (VTE) is typically associated with solid tumors and includes acute superficial and deep venous thrombosis (DVT), pulmonary embolism (PE), and splanchnic vein thrombosis (SPVT) [2]. Cancer patients represent 20% of all patients with DVT and PE, thus the terms cancer-associated thrombosis (CAT) and malignancy-associated coagulopathy (MAC) are commonly used to describe the association among cancer, thrombosis, and coagulation abnormalities in patients with cancer [2,3,4,5].

## 2. Epidemiology

Thromboembolism is the second leading cause of death behind progressive cancer in these patients, having a similar incidence with infection [6]. Fatal PE is three times more common in this group, and overt thrombotic manifestations probably reflect a disease progression, more aggressive phenotypes, or even treatment failure [7,8]. A notable increase in the incidence of CAT has been documented over the past two decades. Interestingly, the 12-month incidence of VTE in people with cancer was reported to be 1% in 1997, increasing to 1.9% in 2004, and to 3.4% in 2017, with no substantial increase in the risk of VTE in patients without cancer during the same period. A possible explanation for this increase could be the improved overall survival of cancer patients, the wider use of chemotherapy, and the introduction of novel targeted therapies. Additionally, the use of CT scans for the diagnosis of thromboembolic events has increased over the past decades, resulting in the identification of PE cases that could otherwise be misdiagnosed [9]. 

Cancer is reported to be evident in approximately 15–25% of all patients with VTE, while patients with active cancer have an 18-fold increased risk for VTE [7,9,10,11,12]. VTE in asymptomatic individuals may be the first sign of an undiagnosed malignancy. In approximately 4% of patients experiencing VTE, a cancer diagnosis is made within one month after the thrombotic event, and the risk is increased by 6% within the first year [7,9]. Accordingly, the risk of VTE is higher during the first period after diagnosis. Cancer is also associated with an increased risk of recurrent thrombosis, estimated to be 15–20% during chemotherapy.

Thrombosis at unusual sites such as mesenteric vein thrombosis, Budd–Chiari syndrome (BCS), and extrahepatic portal vein obstruction (EHPVO) often conceal underlying malignancies. It has been estimated that 25% of patients with splanchnic vein thrombosis (SVT) have a solid tumor, while 8% have a myeloproliferative (MPN) disorder. Moreover, one-third of the patients with EHPVO and almost half of the patients with BCS are diagnosed with MPN disease. Lastly, cerebral vein thrombosis (CVT) could be the first symptom of a solid cancer, mainly a brain tumor, but it can also precede the diagnosis of MPN. It has been reported that JAK2 V617F positivity may occur in patients with SVT and in CVT who do not meet the criteria for MPN [7,9,12]. 

## 3. Risk Factors

**Figure 1:**
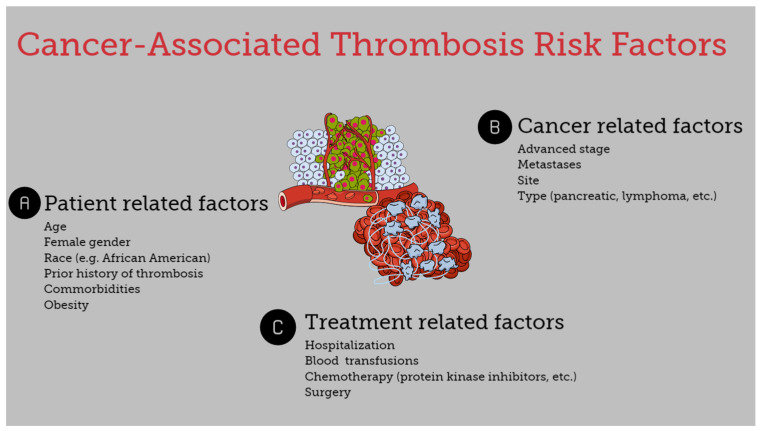
Risk factors for cancer-associated thrombosis.

The risk of thromboembolism in cancer patients is associated with several heterogeneous factors. It is affected by underlying patient-related risk factors, treatment-associated factors, and cancer-specific factors (Figure 1) [7,8,13]. 

### 3.1. Patient-Specific Risk Factors

Age, gender, ethnicity, and genetic and acquired coagulation disorders, along with a history of thrombosis, are important risk factors for the development of thromboembolic disease. Patients >65 years had an increased risk of VTE in a large cohort study of hospitalized cancer patients with neutropenia [14]. Similarly, the risk of thrombosis was higher in surgical patients with pancreatic cancer >60 years compared with younger patients [15]. Regarding race and the risk of CAT, it appears that Asians and Pacific Islanders exhibit a lower risk, whereas African Americans tend to display higher rates of VTE in uterine, ovarian, and breast cancers [10]. Although in most studies, a higher VTE rate has been reported in females, there is no consistency regarding the association between CAT and gender [16]. Certain comorbidities such as obesity substantially increase the risk of VTE, while hospitalization is also associated with increased risk of CAT. Furthermore, cancer-patients with a previous history of thrombosis have a 6- to 7-fold increased risk of recurrent disease, and a family history of VTE is an additional risk factor [7,10,12,17]. Lastly, concurrent prothrombotic risk factors such as FV-Leiden mutation and non-O blood types are also associated with CAT events [17]. Interestingly, patient-related factors such as age, blood type, and VWF and FVIII levels and their correlation probably influence the development of CAT. It seems that with the progression of age, increased VWF and FVIII levels are observed mainly in non-O blood type individuals, participating in the expression of a prothrombotic profile [18].

### 3.2. Treatment-Associated Factors

The use and type of chemotherapy is an important risk factor for VTE development. Chemotherapy is a common reason for hospitalization and is correlated with a 6-fold increase in VTE. However, regardless of the need for hospitalization, chemotherapy is associated with a 2-fold increase in VTE [7,10]. Mulder et al. reported that protein kinase inhibitors, immunotherapy, antiangiogenic therapy, and other targeted therapies were important risk factors for VTE in cancer patients. The authors reported a 12-fold VTE risk in cancer patients during the first six months after diagnosis, and the risk was elevated 23-fold in patients receiving chemotherapy or targeted therapy [9]. Nevertheless, these observations should be interpreted with caution since many of these newer therapeutic agents have substantially contributed to the prolongation of life expectancy in cancer patients. Thus, the higher incidence of VTE in patients receiving these agents may be attributed to the prolonged duration of treatment [6]. 

Surgery is another established risk factor for thrombosis, while the risk of surgery-related VTE is doubled in cancer patients compared with patients without cancer. A prolonged duration of surgery is also associated with an increased risk for CAT. The same applies when surgical re-exploration is needed [19,20,21]. 

### 3.3. Cancer-Specific Risk Factors

Certain types of cancer are associated with a higher rate of thrombotic events. Cancers associated with the highest 6-month VTE incidence include pancreatic cancer, Hodgkin and non-Hodgkin lymphoma, and ovarian cancer. Mesothelioma, brain, lung, kidney, stomach, and hematological malignancies are also associated with an increased risk of VTE. On the other hand, melanoma is associated with the lowest risk, and testicular cancer is also correlated with a low risk for CAT development [7,9,14,22,23,24,25]. The histological subtype of malignancy is also associated with the risk of CAT. For example, thrombotic events are more common in non-small cell lung cancer patients with adenocarcinoma compared with those with squamous cell carcinoma [26], and overall adenocarcinomas demonstrate a higher risk for developing thrombosis [4,7]. Lastly, the stage of the disease also plays a role, as an advanced stage of malignancy and the presence of metastatic disease increase the risk of CAT [10,15,26,27].

## 4. Pathogenesis

**Figure 2:**
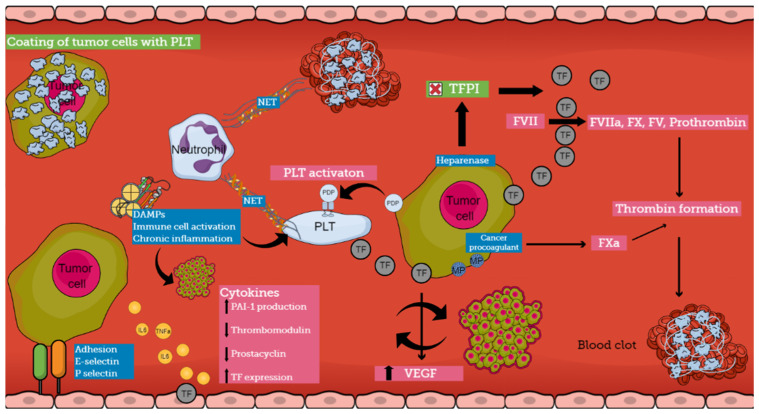
Pathophysiology of cancer-associated thrombosis (abbreviations: TF, tissue factor; PLT, platelet; NET, neutrophil extracellular trap; PDP, podoplanin; TFPI, tissue factor pathway inhibitor; DAMP, damage-associated molecular pattern; MP, microparticle; VEGF, vascular endothelial growth factor; PAI-1, Plasminogen activator inhibitor-1).

The pathogenesis of CAT is multifactorial and involves various mechanisms that are not completely understood (Figure 2). Cancer cells induce a prothrombotic switch in the host hemostatic system and, vice versa, activation of the hemostatic system stimulates tumor growth and dissemination [28,29]. Patients with cancer are in a constant prothrombotic state, presenting abnormalities in all three components of Virchow’s triad [2,4,7,8]. 

Various pathophysiological pathways have been identified in the interplay between different components of the hemostatic system and cancer. Several experimental models demonstrate the development of a prothrombotic microenvironment that promotes tumor growth and establishes a bidirectional relationship between thrombosis and cancer [8]. Thus, cancer cells support clot formation, and clotting proteins support cancer growth [30]. On the other hand, hemorrhagic manifestations also occur, indicating the ability of the tumor to produce fibrinolytic activators, as has been reported in prostate cancer [31]. The understanding of the pathogenesis of CAT is crucial because it can lead to the identification of biomarkers pointing to patients at risk for thrombosis [32].

### 4.1. Direct Mechanisms of CAT

Tumor cells express several procoagulant factors, among which tissue factor (TF) is the most widely studied. TF is constitutively expressed on the surface of tumor cells, while increased levels of expression are associated with an aggressive pattern. Specific genetic alterations in malignant cells lead to the overexpression of this procoagulant molecule. The overall TF production is additionally enhanced by both local (vascular endothelial cells and tumoral stroma) and systematic release (TF-bearing extracellular vesicles) [8]. In the case of slow-release or exposure of TF by the malignant cells, patients may be either asymptomatic or manifest DVT, depending on the rate and quantity of exposure, while massive thrombosis or even severe, life-threatening hemorrhage occurs when large amounts of TF are released in circulation [1]. Tumor-expressed TF not only plays an important role in the generation of thrombin in cancer patients but also influences the expression of VEGF by both malignant and host vascular cells contributing to the progression of a tumor. Thrombin can directly activate cellular receptors promoting intracellular signaling cascades involved in tumor growth and angiogenesis. Recently, several studies have shown that in the presence of procoagulant TF, tumor cells can alter their capacity from indolent to active. Thus, within the tumor microenvironment, procoagulant molecules can influence the fate of naïve cells from a dormant to a fully malignant phenotype [17,28]. Therefore, TF regulates tumor neo-vascularization and metastasis, having a crucial role in the interplay between coagulation and cancer [8,28,30]. 

Other procoagulant molecules expressed by tumor cells include heparanase, cancer procoagulant (CP), and podoplanin (PDP). Heparanase is an enzyme that can upregulate the expression of TF through the disassociation of TFPI, and tumor cells that express heparanase are considered to have increased metastatic potential [1,28,30]. CP is a cysteine protease that demonstrates properties similar to those of activated factor X, but in contrast to TF can activate FX directly and independently of FVII. In patients with acute promyelocytic leukemia (APL), CP is expressed by bone marrow blasts at the onset of the disease and disappears at remission, while the expression of CP reflects the degree of malignant transformation, and its levels are influenced by treatment. Patients who exhibit resistance to therapy tend to express CP continuously, while patients who are responsive to treatment have undetectable levels of CP [1,30]. Podoplanin (PDP) is a platelet-activating and aggregating protein that needs a C-type lectin receptor 2 (CLEC-2) on the surface of platelets for its action. When complete depletion of the receptor occurred in platelets, decreased venous thrombosis was reported in experimental models. Vice versa, when wild-type platelets were infused, increased VTE events were once again observed. The production of PDP has been noted in several cancers such as pancreatic cancer, seminoma, mesothelioma, and pulmonary squamous cell carcinoma, while tumor-associated PDP has been correlated with VTE in patients with brain cancer [33,34,35,36]. Therapies targeting these molecules could be a promising solution in CAT regulation.

Systemic activation of the coagulation cascade in cancer patients is also mediated through procoagulant substances that are expressed on the surface of tumor cells, called microparticles (MPs). These are small membrane vesicles filled with high concentrations of phosphatidylserine and TF. Phosphatidylserine supports the assembly of coagulation complexes on the negatively charged surface of cells. Hematologic malignancies such as APL, AML, and multiple myeloma are characterized by elevated levels of blast cell-derived MPs, while in patients with pancreatic adenocarcinoma, an association between TF-positive MP and VTE complications has been reported [4,17,30,37].

It seems that there is a strong interplay between cancer cells and host normal cells, especially among platelets, leukocytes, and endothelial cells, leading to the induction of a procoagulant phenotype by normal cells. Platelets have a distinct role in CAT mechanisms. Tumor cells can activate platelets, both directly via receptor-mediated mechanisms or indirectly via the secretion of platelet-activating molecules. On the other hand, platelets induce pro-tumoral mechanisms, supporting the tumor microenvironment through the release of growth factors and cytokines [8]. There is a clear correlation between platelet activation and aggregation and metastatic potential. Moreover, it has been hypothesized that cancer-induced platelet aggregation results in the coating of cancer cells with platelets, which helps them escape from natural killer (NK) cell activity [30,38]. 

### 4.2. Indirect Mechanisms of CAT

Cancer cells can activate the procoagulant potential of host vascular cells through the release of soluble mediators. Among the most important soluble mediators are inflammatory cytokines, proangiogenic factors, growth-stimulating factors, and platelet aggregating factors [30]. Tumor cells synthesize and release cytokines and stimulate different cells to activate cytokine networks. Inflammatory cytokines interfere with hemostasis in various ways, as they can downregulate thrombomodulin production, inhibit the release of nitric oxide and prostacyclin by endothelial cells, and stimulate PAI-1 production [4]. TNFa and IL-6 have been reported to induce vWf and TF expression in vascular endothelial cells. Additionally, it is well known that cytokines contribute to the development of DIC; thus, it has been suggested that cancer-related DIC is dependent on cytokines and their ability to modulate coagulation and fibrinolysis. 

Another pathogenetic mechanism of CAT involves the capacity of adhesion molecules to bridge tumor cells with endothelial cells. This leads to the development of localized clots at the vessel wall. E-selectin has been reported to facilitate the connection of colon carcinoma cells (HT-29M) to activated endothelial cells. P-selectin, which is produced by endothelial cells and platelets, also enables binding with malignant cells, even though the ligand on cancer cells has not yet been confirmed. These interactions among cancer cells, platelets, and endothelial cells result in the formation of aggregates that disrupt blood flow and promote vessel occlusion. Furthermore, the interaction among these molecules and mucin from mucinous adenocarcinomas contributes to the formation of platelet microthrombi. Mucins act as ligands for selectins because of their heavy O-linked glycosylation sites, promoting thrombosis in cells [1,4,17]. 

The role of neutrophils in cancer pathogenesis has been well described. Neutrophils are present in large amounts in patients with tumors and release extracellular DNA traps (NETs), which provide activating signals for platelets, enhancing their aggregation and affecting angiogenesis and tumor growth. Recently, it has been suggested that NETs enhance both venous and arterial thrombosis and serve as a platform for direct platelet adhesion and aggregation. Damage-associated Molecular Patterns (DAMPs) are molecules localized within the cell, and they are produced during cell stress or upon cell death from senescent malignant cells. DAMPs can initiate chronic inflammation and immune cell activation. As a result, they contribute to thrombotic events, tumor growth, and tumor survival. Histones and *HMGB1* are DAMPs that have been associated with CAT [4].

Chemotherapy is also associated with hemostatic derangements in CAT. Cytokines seem to be ineffective in regulating anticoagulant mechanisms and fibrinolytic pathways, resulting in an increased thrombotic risk in cancer patients [1,4]. Cisplatin-based chemotherapy promotes thromboembolic events in cancer patients; however, the underlying mechanisms remain unclear. Thalidomide, in combination with chemotherapy, increases the risk of thrombosis in patients with myeloma and renal carcinoma. Anti-angiogenic agents and hormonal therapies have also been associated with increased risk of both venous and arterial thromboembolism, possibly because of their effect on the endothelium. Furthermore, chemotherapeutic agents are hepatotoxic and probably influence the production of natural anticoagulant proteins such as antithrombin and Protein S and C. Moreover, chemotherapy promotes apoptosis in both malignant and host cells, resulting in cytokine release and increased TF expression [1,4,28]. 

Hypoxia is another factor involved in CAT that can indirectly lead to a prothrombotic phenotype. The hypoxic microenvironment created by tumors promotes endothelial perturbation. As a result, increased levels of phospholipase A2 are released, inducing the production of prostaglandins and platelet-activating factor (PAF). Subsequently, PAF activates neutrophils, and the hypoxic microenvironment enables their adhesion to endothelial cells. Moreover, hypoxia causes the exocytosis of Weibel–Palade bodies, facilitating the release of vWf and P-selectin [4,17]. 

Certain oncogenes responsible for the development of the malignancy are associated with CAT. Mutations such as *STK11*, *KRAS*, *CTNNB1*, *CDKN2B*, *KEAP1*, and *MET* are considered predictive of increased occurrence of CAT. Lastly, well-established thrombophilia factors, such as FV Leiden and prothrombin G20210A genes also increase the risk of thrombosis in cancer patients. Recently, an association among a polymorphism of the endothelial protein C receptor (EPCR) and factors V and X with hypercoagulability has been reported in breast cancer patients. Moreover, the homozygous type of the fibrinogen gamma gene (FGG) *rs2066865* has been associated with increased risk for PE in cancer patients [39,40].

## 5. Laboratory Assessment

The development of biomarkers that enable early identification of cancer patients at a high risk of thrombotic events is necessary, as it would enable clinicians to guide thromboprophylaxis measures in these patients [41]. Several biomarkers have been evaluated for their diagnostic value in CAT (Table 1). 

Clinicians have attempted to evaluate the degree of the hypercoagulable state associated with malignancy using conventional tests such as prothrombin time (PT), activated partial thromboplastin time (aPTT), and D-dimers. However, although conventional coagulation tests have a distinct role in the identification of hypocoagulable states, their accuracy in identifying hypercoagulability is limited and not well established. It has been suggested that shortened aPTT is associated with increased thrombotic risk, but because of the demanding pre-analytical process, which is influenced by various factors, this interpretation should be made with caution [42,43]. D-dimers are fibrin degradation products and their presence in circulation is linked to active coagulation. Several studies have shown that D-dimer levels are correlated with an increased risk for thrombosis in cancer patients [12,44,45,46,47,48]. Furthermore, it is suggested that they could be useful biomarkers not only for the prediction of VTE in cancer patients but also for the prediction of recurrent VTE after the cessation of thromboprophylaxis [44,49].

An increased leukocyte count is associated with hypercoagulability in several clinical settings, such as cerebrovascular disease, ischemic heart disease, and MPN disorders [10]. In cancer patients, leukocytosis at the beginning of chemotherapy is an independent risk factor correlated with an increased risk of VTE. Persistent leukocytosis after the first cycle of chemotherapy has also been correlated with increased VTE risk and a causative role of leukocyte count and thrombosis has been suggested. Both increased neutrophil and monocyte counts prior to the initiation of chemotherapy were correlated with elevated risk for thrombosis but, on the contrary, baseline lymphocytosis did not seem to be associated with a higher risk [10,41,50,51]. Accordingly, elevated platelet count before the initiation of chemotherapy has also been correlated with an increased risk for VTE. The increased risk for VTE was maintained during chemotherapy in cancer patients with thrombocytosis, and the cumulative probability of VTE after one year was much higher in patients with elevated platelet counts than in those with lower platelet counts [52]. Platelet-associated biomarkers that could be associated with CAT include Platelet Factor-4 (PF4) and CD40 Ligand. PF4 is released into circulation after platelet activation, and it plays a key role in wound healing and inflammation; however, its role in CAT has not yet been established. CD40 Ligand is also expressed by activated platelets and vascular cells and interacts with soluble CD40L, which is released by activated T-lymphocytes. This interaction is suggested to produce angiogenetic factors such as VEGF, which enhances prothrombotic responses [4].

Thrombin-related molecules such as prothrombin fragment 1 + 2 (F1 + 2) and thrombin-antithrombin complex can also be proven as useful diagnostic biomarkers for the evaluation of CAT. Prothrombin fragment 1 + 2 (F1 + 2) is a by-product of the conversion of prothrombin to thrombin by activated factor X. It can be measured in plasma and is indicative of the coagulation status [4]. It has been shown that cancer patients with elevated F1 + 2 have a 2-fold risk for developing thrombosis, while when the increased levels of F1 + 2 are combined with increased levels of D-dimers, the risk for VTE is even higher [12]. The thrombin generation test (TGT) is another test that can estimate the overall balance between prothrombotic and antithrombotic counterparts of the coagulation cascade. As thrombin is a key enzyme in the coagulation process, a patient’s endogenous thrombin generation potential reflects the contribution of several factors that interfere with blood coagulation. It is a useful tool for the evaluation hypocoagulability or hypercoagulability. An increased level of TGT indicates a pathological coagulation such as in VTE [4,41]. Lastly, the thrombin–antithrombin complex (TAT) is formed by thrombin and antithrombin, and increased TAT levels indicate excess thrombin production; thus, they can be useful biomarkers of the prothrombotic state associated with CAT [4].

Plasminogen activator inhibitor type 1 (PAI-1) also has the potential to be used in CAT. PAI-1 inhibits fibrinolysis, and as a result, increased levels of this marker are associated with thrombosis [53]. There are limited studies evaluating fibrinolysis in patients with CAT. In patients with pancreatic cancer, elevated PAI-1 levels have been associated with a predisposition to VTE [54]. Increased PAI-1 levels have also been reported in patients with glioma compared with healthy controls [55]. Nevertheless, further studies are necessary to determine the role of hypofibrinolysis and specifically the role of PAI-1 in CAT.

Other biomarkers that can be used for the diagnosis and evaluation of CAT include soluble P-selectin and microparticles. Soluble P-selectin (CD62p) is expressed by activated platelets and endothelial cells. It is released from a platelet’s a-granules to its surface and its role is multifactorial. It causes interactions between platelets and the vascular endothelium, enhances cross-linking between platelets and leukocytes, and promotes inflammation upon the release of prο-inflammatory cytokines. The levels of sP-selectin were increased in patients with acute VTE and were associated with an increased risk of VTE in non-cancer patients. In patients with cancer, elevated sP-selectin predicted a 2.6-fold increased risk of developing VTE [4,41,44]. Moreover, patients with distant metastases demonstrate higher sP-selectin levels compared with patients with localized tumors [17]. Microparticles are released by cancer cells, and although elevated MP levels have been found to be associated with a worse prognosis in cancer patients, this is not a consistent finding in the literature [4,50]. However, the main disadvantage of this specific biomarker is that it is not sufficiently standardized [41].

Moreover, viscoelastic methods, such as TEG and ROTEM, are whole blood analyses that provide information for all stages of coagulation and fibrinolytic processes and can be useful in cancer patients [42]. Their utility and predictive value have been documented in sepsis-induced coagulopathy [56], trauma-induced coagulopathy [57], and surgical patients [58]. In patients with cancer, viscoelastic tests can accurately identify hypercoagulability. A correlation between shortened Clotting Formation Time and increased Maximum Clot Firmness with VTE has been reported in patients with cholangiocarcinoma [59]. In 11% of patients with pancreatic cancer and thromboembolism, viscoelastic methods revealed increased a-angle and LY30 [60]. Interestingly, in patients with bone tumors, ROTEM measurements were more representative of the hypercoagulable status compared with conventional tests [61]. In patients who underwent surgery for abdominal malignancies, ROTEM exhibited postoperative hypercoagulability lasting 1–4 weeks [62], and in surgical patients with bone tumors, the hemostatic profile was more adequately managed with the use of ROTEM [63]. In patients with non-small cell lung carcinoma, TEG measurements were indicative of hypercoagulability. Since CAT can precede cancer diagnosis by months, viscoelastic methods may be useful markers for the detection of occult malignancies [42]. Moreover, viscoelastic methods could also be a useful tool for LMWH thromboprophylaxis guidance [64,65].

Another promising thrombogenic marker for the recognition of tumor progression is the von Willebrand factor (VWF). VWF multimers arrange platelet adhesion in vessels and probably participate in both vessel occlusion and tumor progression. VWF, along with the enzyme ADAMTS-13, which inactivates VWF multimers, could be useful markers in CAT management. Elevated VWF levels and reduced ADAMTS-13 activity were higher in patients with VTE, while they were also associated with shorter survival indicating a potential prognostic value of these markers. Moreover, the ratio VWF/ADAMTS-13 has been found to be a useful screening tool for early detection of cancer [66].

Lastly, molecular tests could be also used for the identification of gene mutations involved in the dysregulation of hemostasis in cancer patients. However, molecular tests have not been proven useful in the prediction of CAT so far [4].

Recent research has provided in-depth insight into the pathophysiology of CAT, leading to the development of novel biomarkers that can contribute to improving currently used risk models and decision-making protocols. Traditional biomarkers that have been widely used in CAT patients include D-dimers, PLTs, and WBCs, while sP-selectin and MPs have also been utilized in this setting. Lately, the predictive value of other biomarkers such as VWF, PF4, CD40L, and genetic factors has been evaluated. Moreover, viscoelastic methods provide a dynamic assessment of the hemostatic mechanism and have been gaining ground as a valuable method for evaluating the hypercoagulability associated with CAT. To date, the use of certain biomarkers over others in patients with CAT has not been supported by any guidelines, while further studies are needed to evaluate the value of incorporating all these novel biomarkers in decision-making protocols to guide antithrombotic prophylaxis and therapy.

## 6. Treatment of Cancer-Associated Thrombosis

Given the overlapping altered hemorrhagic and thrombotic pathways in patients with cancer, the management of CAT is challenging [32]. Various anticoagulant agents such as low-molecular-weight heparins (LMWHs), vitamin K antagonists (VKA), and direct oral anticoagulants (DOACs) have been used as a first-line antithrombotic therapy in cancer patients (Table 2).

Historically, LMWH has been the standard treatment of cancer-associated thrombosis since VKAs have not been widely recommended in cancer patients because of the difficulty in PT/INR monitoring, resulting in a high rate of bleeding events. However, a main disadvantage of these agents is their route of administration since they are subcutaneously injected, which causes discomfort and reduces compliance in patients [17,67]. Over the last few years, DOACs have been recognized as an effective alternative to LMWH in patients with cancer, although caution is recommended in patients with gastrointestinal and genitourinary malignancies. DOACs can be orally administered and do not require monitoring, making them a preferable alternative to both LMWHs and VKAs [68,69]. They have also been proven effective in reducing recurrent VTE events compared with LMWH [17,70]. Five pivotal randomized control trials (RCTs) compare individual DOACs with dalteparin, evaluating their safety and efficacy. Specifically, the studies by Agnelli et al. [71] and Raskob et al. [72] and the SELECT-D study [73] and the ADAM VTE study [74] included patients with advanced active cancers and supported the efficacy of DOACs in preventing recurrent VTE events, whereas the smaller CASTA-DIVA study [75] did not establish the non-inferiority of rivaroxaban compared with dalteparin regarding recurrent VTE. However, a higher bleeding risk was observed with DOACs, mainly in patients with gastrointestinal malignancies, in the study by Agnelli et al. (Major Bleeding, MB) and in the SELECT-D study (Clinically Relevant NonMajor Bleeding, CRNMB), where most bleeding events occurred in the upper GI [71,73]. On the contrary, a similar bleeding risk was found in patients receiving apixaban compared with LMWH in the study by Raskob et al., a finding also supported by the ADAM VTE study [72,74]. As there are no studies comparing DOACs on a head-to-head basis, the use of a single DOAC over another cannot be recommended. Nevertheless, apixaban has been included in the current ASCO guidelines as a recommended agent for both initial and extended thromboprophylaxis [76]. The PRIORITY trial compared the safety and efficacy of DOACs to those of dalteparin, enrolling high-risk patients with upper GI, hepatobiliary, and pancreatic cancer [77]. A higher rate of bleeding events (MB and CRNMB) was found in patients using DOACs (rivaroxaban, apixaban) compared with those using dalteparin, suggesting that these specific cancer types are probably more susceptible to bleeding in patients using DOACs. The conflicting results regarding the bleeding risk among the different studies could probably be attributed to the heterogeneity regarding the cancer type and stage; for example, the study by Agnelli et al. and the ADAM VTE study included fewer patients with GI cancer compared with the SELECT-D study and the study by Raskob et al. Therefore, the results of these studies should be interpreted with caution.

The duration of anticoagulant therapy should be at least 3–6 months, while extended treatment may be recommended if the cancer is active or the patient is at high risk for thrombosis. It is still debatable whether thromboprophylaxis should be recommended to all cancer patients because of the high incidence of VTE in these patients, especially when the overall improved survival by their use has not been proven [78]. It is apparent that antithrombotic treatment in cancer patients remains challenging; thus, patient-specific treatment and frequent re-evaluations are needed. Ultimately, the decision to use a DOAC requires careful consideration of the bleeding risk, the cost–benefit, and the patient’s convenience. Furthermore, the use of a specific anticoagulant treatment is often influenced by patient comorbidities that increase the bleeding risk, by the type of cancer, and by factors such as renal or liver dysfunction. VKAs should only be used in patients for whom DOACs and LMWH are unavailable or unsuitable [17,32,79,80,81].

## 7. Risk Assessment Models

There is a broad consensus among investigators that a targeted approach for identifying cancer patients at risk for thrombosis will provide the most optimal risk-benefit ratio for antithrombotic strategies. Despite the well-documented association between cancer and increased risk for thrombosis, clinical studies have not consistently reported improved outcomes with the use of thromboprophylaxis [10,82]. The development of risk assessment models (RAMs) for cancer patients is an attempt to estimate the risk of thrombotic manifestations.

The use of more than a single marker for risk prediction in the form of a risk assessment model is recommended. The first RAM was developed by Khorana et al. [83] for patients undergoing chemotherapy. This model is based on five predictive variables including platelet count, hemoglobin levels, cancer site, body mass index, leukocyte count, and erythropoiesis-stimulating agents (Table 3). Using the aforementioned parameters, a score is calculated for each patient. Based on this score, patients are categorized into three groups as follows: low-, high-, and very high-risk, indicating a VTE risk of 0.5%, 2%, and 7%, respectively [84].

Subsequently, the Khorana score was modified in the Vienna Cancer and Thrombosis Study (CATS) with the addition of two biomarkers, P-selectin and D-dimers, resulting in an improved predictive capacity for VTE [84]. Although both the original Khorana score and the Vienna CATS score have been proven as useful tools, their predictive value may vary based on the population and context in which they are used. Another modified Khorana score is the Protecht score, which includes platinum and gemcitabine-based chemotherapy as risk factors [85]. The Ottawa score is another RAM that aims to identify cancer patients at risk of recurrent thrombosis who could benefit from prolonged anticoagulant therapy [86]. Lastly, Palumbo et al. described another RAM focused on patients with multiple myeloma treated with thalidomide or lenalidomide, to identify high-risk patients [87]. The need for using more than one parameter for the prediction of CAT is also highlighted in the guidelines released by the American Society of Clinical Oncology [86]. Thus, it is advisable to incorporate several clinical and biological markers into scoring systems [30,88].

## 8. Current Guidelines

Several guidelines by various hematology and oncology scientific societies have been published for the prevention of VTE in cancer patients. The American Society of Clinical Oncology (ASCO), the American Society of Hematology (ASH), the National Comprehensive Cancer Network (NCCN), the International Initiative on Thrombosis and Cancer (ITAC), and the European Society of Medical Oncology (ESMO) have published their own guidelines on the primary prevention of VTE in cancer patients (Table 4) [76,79,89,90,91,92].

There is general consensus regarding the need for risk stratification, the increased risk for VTE in patients with advanced disease, and the importance of evidence-based approaches for primary thromboprophylaxis. However, there is a high heterogeneity in the recommended guidelines by these different organizations [6]. The ASH guidelines reported that although the use of thromboprophylaxis decreased the risk of PE, the risk of DVT and the overall mortality resulting from VTE were not affected. Thromboprophylaxis is recommended for hospitalized cancer patients and critically ill cancer patients without any high risk for bleeding [93,94,95,96,97]. Thromboprophylaxis is also recommended in all cancer patients undergoing surgery. A four-week extended thromboprophylaxis is recommended following abdominal or pelvic surgery in high-risk patients for VTE, given that they are not at high risk for bleeding [98].

Interestingly, a meta-analysis of randomized trials involving acutely ill patients requiring hospitalization trials reported that the use of thromboprophylaxis did not influence the risk of VTE compared to a placebo [93,94,95,99]. It should be noted though that many VTE cases are asymptomatic and that almost 45% of VTE events occur after patient discharge; therefore, it is not easy to estimate to which extent thromboprophylaxis prevents VTE [6]. Lastly, no significant differences in terms of bleeding, mortality, or thromboembolic outcomes have been reported between different heparin agents (LMWH, UFH, and fondaparinux) [100].

## 9. Conclusions

CAT is a common complication in cancer patients that affects their quality of life, while it has a negative impact on the survival of these patients. However, thrombotic events in cancer patients may be preventable in many cases. Therefore, the use of RAMs for the stratification of cancer patients according to their propensity to develop VTE along with the development of accurate biomarkers should be the focus of future research, for the development of successful strategies to reduce the overall rate of these devastating complications.

## Figures and Tables

**Table 1:** Diagnostic tests in cancer-associated thrombosis.

Conventional Tests	
PT/INR	Normal
aPTT	Normal or shorter * (* not established)
D-Dimers	Elevated (CATS score)
White blood count	Normal or elevated (not established)
Markers of hypercoagulability	
F1 + 2 prothrombin fragment	Elevated (not used as routine screening)
Thrombin generation test	Elevated (not used as routine screening)
Thrombin antithrombin complex	Elevated (not used as routine screening)
Markers of impaired fibrinolysis	
Plasminogen activator inhibitor-1	Elevated (not used as routine screening)
Novel biomarkers	
Soluble CD62P	Elevated (CATS score)
Microparticles	Elevated (not standardized)
Platelet Factor-4	Elevated (not established)
CD40 Ligand	Elevated (not established)
VWF/ADAMTS 13 ratio	Elevated (not established)
Molecular tests	Uncertain
Viscoelastic methods (POC)	
ROTEM	Shorter CT, increased MCF, increased a-angle
TEG	Shorter R and K time, increased MA

Abbreviations: PT, prothrombin time; INR, International Normalized Ratio; aPTT, activated partial thromboplastin time; ROTEM, rotational thromboelastometry; TEG, thromboelastography; CT, clotting time; MC, Maximum Clot Firmness; MA, maximum amplitude.

**Table 2:** Treatment options for cancer-associated thrombosis.

Therapeutic Agent	Advantages	Disadvantages
Vitamin K antagonists		PT/INR monitoring. High bleeding risk.
LMWHs	Compared to Vit K antagonists Reduced recurrent thrombotic events without significant increase in major bleeding events. Fewer drug-to-drug interactions. Dependable pharmakocinetics. Short half-life time.	Route of administration is not preferable by patients. High cost.
DOACs	Compared to LMWH Patient compliance due to per os use. Patience preference due to the route of administration. Reduced recurrent thrombotic events.	Compared to LMWH Increase in non-major bleeding events. Increased gastrointestinal bleeding events in patients with gastrointestinal cancer. Drug-to-drug interactions. Caution in patients with impaired renal and liver function.

Abbreviations: LMWH, low-molecular-weight heparin; DOAC, direct oral anticoagulants.

**Table 3:** Khorana risk assessment model.

Parameters		Points
Tumor type	Very high-risk tumor (pancreatic, stomach)	2
	High-risk tumor (bladder, gynecological, lung, lymphoma)	1
	Other type of tumor	0
Thrombocytosis (≥350 × 10^9^/L)		1
Elevated pre-chemotherapy white blood count (>11 × 10^9^/L)		1
High Body Mass Index (≥35 kg/m^2^)		1
Low hemoglobin level (<10 g/Dl)		1
Use of erythropoietin stimulating agent		1
Low Risk (0–1 points): no prophylaxis is recommended. Intermediate risk (2 points): consider prophylaxis. High risk (≥3 points): prophylaxis indicated.		

**Table 4:** Current guidelines for the management of cancer-associated thrombosis.

Scientific Society	Initial Treatment Recommendations	Long Term Treatment Recommendations
ASCO [79]	Recommended agents: UFH, LMWH, fondaparinux, rivaroxaban.	Recommended agents: LMWH, edoxaban, rivaroxaban. - Duration > 6 months.
ASCO [76]	Recommended agents: UFH, LMWH, fondaparinux, rivaroxaban, apixaban.	Recommended agents: LMWH, edoxaban, rivaroxaban, *apixaban*. - Duration > 6 months.
NCCN [92]	Recommended agents: Apixaban, rivaroxaban, edoxaban (in patients without gastroesophageal lesions). LMWH (in patients with gastroesophageal lesions).	
ASH [89]	Recommended agents: apixaban, rivaroxaban or LMWH - DOACs are recommended over Vit K antagonists and LMWH as short-term treatment (3–6 months). - DOACs should be administrated with caution in patients with GI cancers.	Recommended agents: LMWH, DOACs - Duration > 6 months.
ESMO [93]	Recommended agents: UFH, LMWH, apixaban, rivaroxaban, fondaparinux for initial acute phase treatment. - LMWH is recommended over UFH and fondaparinux. - UFH is a considerable option for patients with renal dysfunction (CrCl < 30 mL/min).	Vitamin-K antagonists are not recommended.
ITAC [94]	Recommended agents: LMWH, DOACs when CrCl >/= 30 mL/min. UFH when LMWH and DOAC are contraindicated. - DOACs are not recommended in patients gastrointestinal or genitourinary bleeding.	Should be based on customized evaluation.

Abbreviations: ASCO, The American Society of Clinical Oncology; NCCN, National Comprehensive Cancer Network; ASH, American Society of Hematology; ESMO, European Society of Medical Oncology; ITAC, International Initiative on Thrombosis and Cancer; UFH, unfractioned heparin; LMWH, low-molecular-weight heparin; DOAC, direct oral anticoagulants.
